# Immunization with genetically attenuated P52-deficient *Plasmodium berghei *sporozoites induces a long-lasting effector memory CD8^+ ^T cell response in the liver

**DOI:** 10.1186/1476-8518-9-6

**Published:** 2011-10-17

**Authors:** Bruno Douradinha, Melissa van Dijk, Geert-Jan van Gemert, Shahid M Khan, Chris J Janse, Andy P Waters, Robert W Sauerwein, Adrian JF Luty, Bruno Silva-Santos, Maria M Mota, Sabrina Epiphanio

**Affiliations:** 1 Unidade de Malaria, Instituto de Medicina Molecular, Universidade de Lisboa, Av Professor Egas Moniz, Lisboa, 1649-028, Portugal; 2 Instituto Gulbenkian de Ciencia, Rua da Quinta Grande, 6, Oeiras, 2780-156, Portugal; 3 Department of Parasitology, Leiden University Medical Center, P.O. Box 9600, Leiden, 2300 RC, The Netherlands; 4 Department of Medical Microbiology, University Medical Centre, P.O. Box 9101, Nijmegen, 6500 HB, The Netherlands; 5 Unidade de Imunologia Molecular, Instituto de Medicina Molecular, Universidade de Lisboa, Av Professor Egas Moniz, Lisboa, 1649-028, Portugal; 6 Departamento de Ciências Biológicas, Universidade Federal de São Paulo, Rua Professor Artur Riedel, 275, Diadema, 09972-270, Brasil; 7 Fondazione Ri.Med, Piazza Sett'Angeli 10, Palermo, 90134, Italy; 8 Faculty of Earth and Life Sciences, VU University Amsterdam, De Boelelaan 1085-1087, Amsterdam, 1081 HV, Amsterdam, The Netherlands; 9 Division of Infection and Immunity, Institute of Biomedical Life Sciences & Wellcome Centre for Molecular Parasitology, Glasgow Biomedical Research Centre, Sir Graeme Davies Building, 120 University Place, G12 8TA, Glasgow, UK

## Abstract

**Background:**

The induction of sterile immunity and long lasting protection against malaria has been effectively achieved by immunization with sporozoites attenuated by gamma-irradiation or through deletion of genes. For mice immunized with radiation attenuated sporozoites (RAS) it has been shown that intrahepatic effector memory CD8^+ ^T cells are critical for protection. Recent studies have shown that immunization with genetically attenuated parasites (GAP) in mice is also conferred by liver effector memory CD8^+ ^T cells.

**Findings:**

In this study we analysed effector memory cell responses after immunization of GAP that lack the P52 protein. We demonstrate that immunization with *p52*^-^GAP sporozoites also results in a strong increase of effector memory CD8^+ ^T cells, even 6 months after immunization, whereas no specific CD4^+ ^effector T cells response could be detected. In addition, we show that the increase of effector memory CD8^+ ^T cells is specific for the liver and not for the spleen or lymph nodes.

**Conclusions:**

These results indicate that immunization of mice with *P. berghei p52*^-^GAP results in immune responses that are comparable to those induced by RAS or GAP lacking expression of UIS3 or UIS4, with an important role implicated for intrahepatic effector memory CD8^+ ^T cells. The knowledge of the mediators of protective immunity after immunization with different GAP is important for the further development of vaccines consisting of genetically attenuated sporozoites.

## Findings

Immunization studies using live radiation-attenuated sporozoites (RAS) demonstrated full protection against subsequent challenge with infectious plasmodial sporozoites in mice, in non-human primates, and in humans [reviewed in [1]]. This protection is now known to be mediated by complex mechanisms involving both antibody and T cell responses [reviewed in [2]]. The long-lasting RAS-induced sterile immunity in mice is characterized by the establishment of a CD44^high^CD45RB^low^CD62L^low ^subset of memory CD8^+ ^T cells (but not of CD4^+ ^T cells) in the liver but not in the spleen [3,4]. Recently, it has been shown that genetically attenuated parasites (GAP), lacking conserved sporozoite-specific genes that are important for development inside the hepatocyte, can induce partial or complete protective immunity in rodent models of malaria [5-10]. We have previously characterized the immunization potential of *P. berghei p52*^-^GAP [7,9] that do not express the sporozoite-specific microneme protein P52 (PBANKA_100220; also known as P36p) [11]. Here, we sought to quantify memory lymphocytes, elicited by immunizations with *p52*^-^GAP, which may play a role in maintenance in the long-lasting immunity conferred by these attenuated parasites. We show that the long-lasting protection elicited by this GAP is coincident with the presence and persistence of an expanded population of CD8^+ ^effector memory T cells found only in the liver.

### High levels of CD8^+ ^effector memory T cells are maintained for up to 6 months exclusively in the livers of *p52*^- ^GAP-immunized mice

We have recently shown that BALB/c mice immunized with *p52*^-^GAP are protected when challenged with infectious *P. berghei *sporozoites 6 months later, without requiring additional boosts [9]. Immunological memory in RAS-immunized C57BL6 mice is associated with the establishment of effector memory CD8^+ ^T cells found in the liver, but not in the spleen [3,4]. Similar results were observed for *P. berghei *double knockout *uis3*^-^*/uis4*^-^GAP in C57BL6 mice [12] and *P. yoelii uis4*^-^GAP in BALB/c mice [13]. To determine whether similar cells are elicited by *p52*^-^GAP, we quantified effector memory CD8^+ ^T cells in different organs of GAP-immunized BALB/c mice (this study was carried out in strict accordance with the recommendations of both the Animal Experiment Committees governed by section 18 of the Experiments on Animals Act and registered by the Dutch Inspectorate for Health, Protection and Veterinary Public Health (Ministry of Health, Welfare and Sport), and the Portuguese official Veterinary Directorate, which complies with the Portuguese Law (Portaria 1005/92); the Dutch and Portuguese Experiments on Animal Act strictly comply with the European Guideline 86/609/EEC and follow the Federation of European Laboratory Animal Science Associations guidelines and recommendations concerning laboratory animal welfare. In The Netherlands, all animal experiments were approved by the Animal Experiments Committee of the LUMC (ADEC). In Portugal, all animal experiments were approved by the Portuguese official veterinary department for welfare licensing and the Instituto Gulbenkian de Ciencia Animal Ethics Committee). Six-week old female BALB/c mice (Instituto Gulbenkian de Ciência, Oeiras, Portugal) were immunized intravenously with 50,000 *p52*^- ^*P. berghei *sporozoites (ANKA strain, expressing GFP [7], obtained by hand dissection of infected mosquitoes [14]). *p52*^-^GAP-immunized mice were sacrificed at different times post-immunization (10 days, 1 and 6 months) to collect livers, spleens and lymph nodes, from which non- parenchymal cells were isolated as described previously [3,4,15]. In parallel, mice immunized identically were challenged with 10,000 infectious *P. berghei *ANKA sporozoites, confirming full protection against subsequent infection, at all the time points (10 days, 1 and 6 months) memory cells were analysed (data not shown).

For fluorescence-associated cell sorting (FACS) analyses, lymphocytes were incubated with fluorochrome-conjugated antibodies for 15 minutes on ice, washed and resuspended for FACS acquisition on a flow cytometer FACSCalibur (BD Biosciences). For each group of mice, a representative pool of cells was analyzed. Memory CD8^+ ^T cells were quantified using the combination of CD8-PerCP-Cy5/CD44-APC/CD62L-FITC antibodies (Pharmingen).

**Figure 1:**
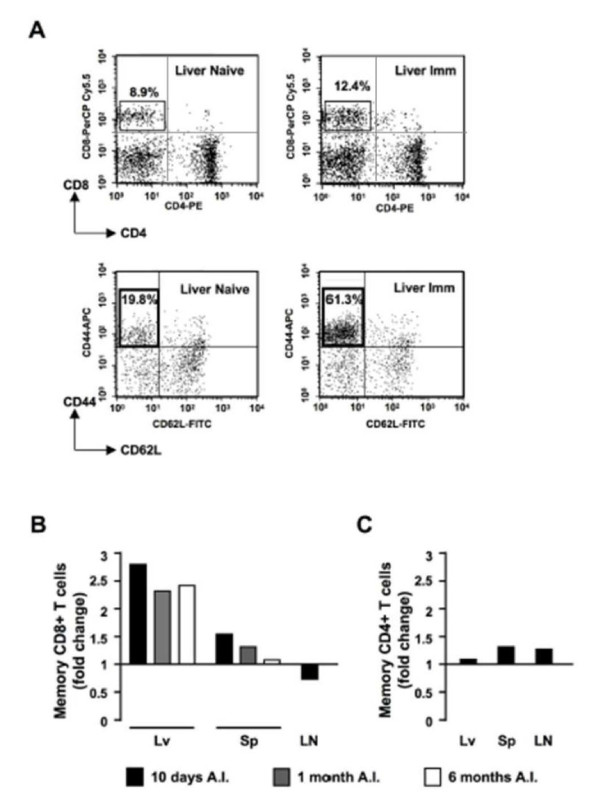
**Effector memory CD8^+ ^and CD4^+ ^T cells in naïve and *p52*^- ^immunized mice**. For each analysed organ, cell populations were defined as either CD8^+ ^or CD4^+ ^T cells, and subpopulations of effector memory cells within those populations were defined as CD44^high^CD62L^low^. (A) Flow cytometry analysis of CD8^+^CD44^high^CD62L^low ^memory T cells in the liver of naïve (left panels) or immunized (Imm, right panels) mice 10 days after immunization with *p52*^- ^sporozoites. Although the levels of CD8^+ ^T cells are similar in both naïve and Imm mice (8.9% versus 12.4%, upper panels), the percentage of CD8^+ ^effector memory cells is much higher in Imm than in naïve mice (61.3% versus 19.8%, lower panels). Values refer to percentage of cells in the respective quadrant. (B) Relative proportion (fold increase) of CD8^+ ^effector memory T cells in naïve and immunized mice (n = 3 per group) in liver (Lv), spleen (Sp) and lymph nodes (LN) at different time points after immunization (A.I.). Effector memory CD8+ T cells were quantified as shown in (A) and results are displayed as fold increase in immunized mice over naive mice. (C) Relative proportion of CD4^+^CD44^high^CD62L^low ^(effector) memory T cells in the same immunized and naïve mice as shown in (B). These cells were quantified as described in (B) from mice at 10 days after immunization. FACS analyses were performed on pooled cells from three animals within the same group, and 100,000 to 200,000 events per each phenotype studied were analysed.

The numbers of CD8^+ ^effector memory T cell (CD44^high^CD62L^low^) increased nearly three-fold in the livers of immunized versus naïve mice when measured 10 days post-immunization (Figure 1A, B). At 1 month post-immunization, the CD8^+^CD44^high^CD62L^low ^cell population was still ~2.5-fold higher in the livers of immunized versus naïve mice, and remained at the same level 6 months post-immunization (Figure 1B).

In the spleens of *p52*^-^GAP-immunized mice, only a modest increase (~1.5 fold) in the number of CD8^+ ^effector memory T cells was observed 10 days post-immunization (Figure 1B), returning to basal levels 1 month post-immunization (Figure 1B).

The initial site of induction of memory CD8^+ ^T cells found in the liver after immunization with attenuated sporozoites is unclear. It has been suggested that it occurs in the liver itself or that such cells are induced in draining lymph nodes, from where they migrate to the liver following sporozoite challenge [16]. We therefore quantified effector memory CD8^+ ^T cells in the lymph nodes of non-immunized and *p52*^-^GAP-immunized mice, 10 days post-immunization. No increase in effector memory CD8^+ ^T cells was observed in the immunized mice (Figure 1B), suggesting that the liver rather than lymph nodes is probably the principal site of induction of memory T cells following *p52*^-^GAP immunization.

### CD4^+ ^effector memory T cells are not induced after immunization with *p52*^- ^sporozoites.

Effector memory CD4^+ ^T cells were quantified by FACS as described for CD8^+ ^T cells, with the following conjugated antibodies: CD4+ PE/CD44-APC/CD62L-FITC (Pharmingen). In contrast to effector memory CD8^+ ^T cells, there was no marked increase in the number of CD4^+ ^effector memory T cells (defined as CD44^high^CD62L^low^) in any of the organs examined after immunization (Figure 1C)

Protection against chronic infections like malaria requires robust responses by the lymphocyte arm of the adaptive immune system, and relies on induction and maintenance of immunological memory. In our studies with *p52*^-^GAP, maintenance of protective immune responses after the first immunization in BALB/c mice required no additional boosting by challenge infections, for example, and mice remained fully protected for up to 6 months [9]. We found that maintaining protective immunity beyond this 6 months period, however, does require *de novo *antigen presentation via, for example, additional challenge infection with sporozoites. Renewed antigen exposure thus seems to be necessary to maintain full protective immune responses over prolonged periods after the initial GAP immunization. RAS-immunized mice indeed require the presence of antigen to maintain protective immunity [17], through re-stimulation of intrahepatic memory T cells [4]. Such re-stimulation can comprise challenge infection with wild-type parasites or may result from the presence of persisting antigen [reviewed in 18]. Persistence of plasmodial antigen can derive from either residual parasites in the liver or in the form of circulating parasite proteins or peptide-MHC complexes. It has been suggested that apoptotic infected hepatocytes could provide plasmodial antigens to professional APC [reviewed in [2,18]]. These would consequently migrate from the liver and act like an antigen depot, explaining why mice treated with drugs to clear intrahepatic parasite load can still be protected [17-19]. Also, antigenic load supplied by the different immunization strategies appears to be a critical factor; a high dose of antigen would mean a larger antigen depot, therefore inducing a more long-lasting protection [reviewed in [2,18]]. We found that, in *p52*^-^GAP-immunized mice, the population of CD8^+ ^effector memory T cells in the liver, at 10 days post-immunization, is greater than that observed at later time points, most probably indicating depletion or exhaustion of the antigen depot. A very recent work states that antigen persistence occurs in live- and RAS- immunized mice even upon drug treatment to clear the liver parasites [17]. In these conditions, the development of a CD8^+ ^T cell response was also efficient, but not when heat-killed sporozoites were used, indicating that only metabolically active parasites can act as source of antigen. Therefore, as seems to be the case for RAS-induced protection [17-19], *p52*^-^GAP-induced protective immune responses appear to depend on continuous antigen presentation provided by a persisting antigen depot. In the study presented here, perhaps not unexpectedly, we observed similarities between the immune responses induced by GAP and RAS. As already reported for RAS [3,4], *P. berghei uis3*^-^*/uis4*^- ^GAP [12] and *P. yoelii uis4*^-^GAP [13], *p52*^-^GAP immunization elicits effector memory CD8^+ ^T cells solely in the liver and not in the spleen or lymph nodes, indicating that organ-specific responses in the liver are probably crucial for the establishment of long-lasting protection in this system. Although priming of CD8+ T cells against plasmodial sporozoites' antigens occurs also in skin-draining lymph nodes, hepatocytes can also present plasmodial antigens via MHC classe I [reviewed in [18]]. The later would be more efficient, since a broader range of antigens (from both sporozoite and liver developing plasmodial schizont) would be presented to the immune system. Also, the vast majority of sporozoites reach the liver shortly after inoculation, strengthening the hypothesis that most of the induction of immune cells occurs in the liver. Regarding effector memory CD4^+ ^T cells, their numbers remain unchanged following *p52*^-^GAP immunization, as has been found in RAS-immunized mice [3], implying no major role for these cells in the protection conferred by attenuated parasites. Our results, together with those of others [3,4,12,13,18,20], strongly emphasize the role of the liver, and particularly of intrahepatic effector memory CD8^+ ^T cells, in the maintenance of the sterile immunity conferred by attenuated plasmodial sporozoites.

## List of abbreviations used

RAS: Radiation-attenuated sporozoites; GAP: Genetically-attenuated parasites; uis: Upregulated in sporozoites; CD: Cluster of differentiation; FACS: Fluorescence-associated cell sorting; MHC: Major Histocompatibility Complex; APC: Antigen Presenting Cell

## Competing interests

The authors declare that they have no competing interests.

## Authors' contributions

BD performed mice liver stage infection and immunizations assays, organs collection, non-parenchymal cells extraction and staining, FACS, data analysis and participated both in the study design and manuscript draft. MRvD, SKM, CJJ, APW and RWS participated in the study design. GJvG reared and infected the mosquitoes with wild-type and *p52*^- ^*P. berghei *parasites used in this work. AJFL coordinated GJvG work and participated in the study design. BS-S co-supervised BD, performed FACS, data analysis, designed the study and participated in its coordination. MMM supervised BD and SE, conceived and coordinated the study, participated in its design and manuscript preparation. SE co-supervised BD, optimized and performed the technique for extraction and purification of non-parenchymal cells and organ collection, participated in both design and coordination of the study and manuscript preparation. All authors read and approved final manuscript.

## References

[B1] Hoffman SL Billingsley PF James E Richman A Loyevsky M Li T Chakravarty S Gunasekera A Li M Stafford R Development of a metabolically active, non-replicating sporozoite vaccine to prevent Plasmodium falciparum malaria Hum Vaccin 2010 6 1 10.4161/hv.6.1.10396 19946222

[B2] Doolan DL Martinez-Alier N Immune response to pre-erythrocytic stages of malaria parasites Curr Mol Med 2006 6 2 169 185 10.2174/156652406776055249 16515509

[B3] Guebre-Xabier M Schwenk R Krzych U Memory phenotype CD8(+) T cells persist in livers of mice protected against malaria by immunization with attenuated *Plasmodium berghei *sporozoites Eur J Immunol 1999 29 12 3978 3986 10.1002/(SICI)1521-4141(199912)29:12<3978::AID-IMMU3978>3.0.CO;2-0 10602007

[B4] Berenzon D Schwenk RJ Letellier L Guebre-Xabier M Williams J Krzych U Protracted protection to *Plasmodium berghei *malaria is linked to functionally and phenotypically heterogeneous liver memory CD8+ T cells J Immunol 2003 171 4 2024 2034 12902507 10.4049/jimmunol.171.4.2024

[B5] Mueller AK Camargo N Kaiser K Andorfer C Frevert U Matuschewski K Kappe SH *Plasmodium *liver stage developmental arrest by depletion of a protein at the parasite-host interface Proc Natl Acad Sci USA 2005 102 8 3022 3027 10.1073/pnas.0408442102 15699336 PMC548321

[B6] Mueller AK Labaied M Kappe SH Matuschewski K Genetically modified *Plasmodium *parasites as a protective experimental malaria vaccine Nature 2005 433 7022 164 167 10.1038/nature03188 15580261

[B7] van Dijk MR Douradinha B Franke-Fayard B Heussler V van Dooren MW van Schaijk B van Gemert GJ Sauerwein RW Mota MM Waters A Genetically attenuated, P36p-deficient malarial sporozoites induce protective immunity and apoptosis of infected liver cells Proc Natl Acad Sci USA 2005 102 34 12194 12199 10.1073/pnas.0500925102 16103357 PMC1189305

[B8] Falae A Combe A Amaladoss A Carvalho T Menard R Bhanot P Role of Plasmodium berghei cGMP-dependent protein kinase in late liver stage development J Biol Chem 2010 285 5 3282 3288 10.1074/jbc.M109.070367 19940133 PMC2823412

[B9] Douradinha B van Dijk MR Ataide R van Gemert GJ Thompson J Franetich JF Mazier D Luty AJ Sauerwein R Janse CJ Genetically attenuated P36p-deficient Plasmodium berghei sporozoites confer long-lasting and partial cross-species protection Int J Parasitol 2007 37 13 1511 1519 10.1016/j.ijpara.2007.05.005 17604034

[B10] Labaied M Harupa A Dumpit RF Coppens I Mikolajczak SA Kappe SH Plasmodium yoelii sporozoites with simultaneous deletion of P52 and P36 are completely attenuated and confer sterile immunity against infection Infect Immun 2007 75 8 3758 3768 10.1128/IAI.00225-07 17517871 PMC1951999

[B11] Ishino T Chinzei Y Yuda M Two proteins with 6-cys motifs are required for malarial parasites to commit to infection of the hepatocyte Mol Microbiol 2005 58 5 1264 1275 10.1111/j.1365-2958.2005.04801.x 16313615

[B12] Jobe O Lumsden J Mueller AK Williams J Silva-Rivera H Kappe SH Schwenk RJ Matuschewski K Krzych U Genetically attenuated Plasmodium berghei liver stages induce sterile protracted protection that is mediated by major histocompatibility complex Class I-dependent interferon-gamma-producing CD8+ T cells J Infect Dis 2007 196 4 599 607 10.1086/519743 17624847 PMC3594113

[B13] Trimnell A Takagi A Gupta M Richie TL Kappe SH Wang R Genetically attenuated parasite vaccines induce contact-dependent CD8+ T cell killing of Plasmodium yoelii liver stage-infected hepatocytes J Immunol 2009 183 9 5870 5878 10.4049/jimmunol.0900302 19812194

[B14] Ozaki LS Gwadz RW Godson GN Simple centrifugation method for rapid separation of sporozoites from mosquitoes J Parasitol 1984 70 5 831 833 10.2307/3281779 6150971

[B15] Epiphanio S Mikolajczak SA Goncalves LA Pamplona A Portugal S Albuquerque S Goldberg M Rebelo S Anderson DG Akinc A Heme oxygenase-1 is an anti-inflammatory host factor that promotes murine plasmodium liver infection Cell Host Microbe 2008 3 5 331 338 10.1016/j.chom.2008.04.003 18474360

[B16] Krzych U Schwenk J The dissection of CD8 T cells during liver-stage infection Curr Top Microbiol Immunol 2005 297 1 24 10.1007/3-540-29967-X_1 16265901

[B17] Cockburn IA Chen YC Overstreet MG Lees JR van Rooijen N Farber DL Zavala F Prolonged Antigen Presentation Is Required for Optimal CD8+ T Cell Responses against Malaria Liver Stage Parasites PLoS Pathog 2010 6 5 e1000877 10.1371/journal.ppat.1000877 20463809 PMC2865532

[B18] Douradinha B Doolan DL Harnessing immune responses against *Plasmodium *for rational vaccine design Trends Parasitol 2011 27 274 83 10.1016/j.pt.2011.01.002 21531627

[B19] Scheller LF Azad AF Maintenance of protective immunity against malaria by persistent hepatic parasites derived from irradiated sporozoites Proc Natl Acad Sci USA 1995 92 9 4066 4068 10.1073/pnas.92.9.4066 7732032 PMC42103

[B20] Schmidt NW Podyminogin RL Butler NS Badovinac VP Tucker BJ Bahjat KS Lauer P Reyes-Sandoval A Hutchings CL Moore AC Memory CD8 T cell responses exceeding a large but definable threshold provide long-term immunity to malaria Proc Natl Acad Sci USA 2008 105 37 14017 14022 10.1073/pnas.0805452105 18780790 PMC2544571

